# Cognitive and behavioural processes in adolescents with social anxiety disorder

**DOI:** 10.1016/j.brat.2023.104416

**Published:** 2023-11

**Authors:** Eleanor Leigh, Ray Percy, David M. Clark, Cathy Creswell, Polly Waite

**Affiliations:** a Department of Experimental Psychology, University of Oxford, UK; b School of Psychology and Clinical Language Sciences, University of Reading, UK; c Department of Experimental Psychology and Department of Psychiatry, University of Oxford, UK

## Abstract

**Background:**

A better understanding of the processes that maintain social anxiety disorder (SAD) in adolescents could improve treatment outcomes. This study aimed to establish whether cognitive and behavioural processes known to be important in the maintenance of adult SAD are observed in adolescent populations and whether they are specific to SAD.

**Methods:**

We recruited three groups of adolescents (12-18y): (1) 90 adolescents with a SAD diagnosis, (2) 58 adolescents with an anxiety disorder that was not SAD, and (3) 45 community-based adolescents. Participants completed measures of negative social cognitions, social attitudes, safety behaviours, self-focused attention, and social anxiety, anxiety, and depression symptoms.

**Results:**

The clinical SAD sample endorsed higher levels of negative social cognitions, attitudes, and safety behaviours compared to both control groups. Self-focused attention was higher in the clinical SAD sample compared to the anxiety clinical control group but not compared to the community control group.

**Conclusions:**

This study provided evidence of SAD-specific mechanisms including negative social cognitions, attitudes and safety behaviours in adolescents. The study did not provide evidence of disorder-specific mechanisms of self-focused attention but this may have been due to methodological limitations. These findings indicate avenues for further research and point to the potential value of Cognitive Therapy for the treatment of adolescent SAD.

## Highlights

• We examined social anxiety-related cognitive and behavioural processes in adolescence.
• Youth with clinical SAD, non-SAD anxiety disorder, and an unselected community group were compared.
• Negative cognitions, attitudes, and safety behaviours were higher in the clinical SAD group vs. control groups.
• Self-focused attention was higher in clinical SAD vs. anxiety control group, but not when compared to community control.
• Findings point to the potential value of adolescent SAD treatments that target these processes.

## Funding information

Eleanor Leigh was funded by a 10.13039/100010269Wellcome Trust Clinical Research Training Fellowship (102176/Z/13/Z) and is now funded by a 10.13039/501100000265MRC Clinician Scientist Fellowship (MR/W02389X/1). Polly Waite was funded by an 10.13039/501100000272NIHR Postdoctoral Research Fellowship (PDF-2016-09-092) and is now funded by an 10.13039/501100000272NIHR Development and Skills Enhancement award (NIHR302843). David M Clark was funded by the 10.13039/100010269Wellcome Trust (WT069777). Cathy Creswell was funded by an 10.13039/501100000272NIHR Research Professorship (RP-2014-04-018) and receives support from the Oxford and Thames Valley 10.13039/501100000272NIHR Applied Research Collaboration and the Oxford Health 10.13039/501100000272NIHR Biomedical Research Centre. The study was sponsored by the 10.13039/501100000839University of Reading. For the purpose of Open Access, the author has applied a CC BY public copyright licence to any Author Accepted Manuscript version arising from this submission.

## 1 Introduction

Anxiety disorders are among the most common psychological disorders in young people, with global community prevalence rates of around 6.5% (Polanczyk, Salum, Sugaya, Caye, & Rohde, 2015). The most common anxiety disorder in adolescents is social anxiety disorder (SAD), with lifetime prevalence rates of around 7% (Fehm, Pelissolo, Furmark, & Wittchen, 2005). SAD is characterised by persistent and disproportionate fear of social situations where embarrassment may occur. It is associated with an increased risk of other mental health problems, including other anxiety and depressive disorders, substance abuse and psychosis (Beesdo et al., 2007; Chartier, Walker, & Stein, 2003; Schutters et al., 2012). Adolescents with SAD also have an increased risk of leaving school early and educational underachievement (Vilaplana-Pérez et al., 2020). Furthermore, it has the lowest natural recovery rate of all anxiety disorders (Bruce et al., 2005).

We have effective treatments for SAD in adults. For example, Cognitive Therapy for SAD (CT-SAD) achieves high recovery rates (of up to 84%; Mayo-Wilson et al. (2014)) and is one of the first-line treatments recommended in the National Institute for Health and Care Excellence (2013) guidelines. The treatment was designed to reverse the specific cognitive and behavioural processes invoked in the cognitive model of Clark and Wells (1995). According to this model, social anxiety is driven by excessively negative beliefs about social interactions, which are maintained by self-focused attention, maladaptive safety behaviours, and negative self-imagery. There is substantial empirical support for the model in adult 10.13039/501100009237SAD (see Leigh and Clark (2018) for a summary).

SAD typically starts in adolescence (median 13 years), with most people developing the condition before they reach their twenties (Beesdo-Baum et al., 2012; Kessler et al., 2005). SAD-specific treatments such as those of Masia-Warner, Fisher, Shrout, Rathor, and Klein (2007) and Beidel et al. (2007) have been developed and show promising outcomes. However, these treatments do not show a strong focus on the psychological processes specified in the Clark and Wells’ model, and direct evidence of superiority to generic CBT is currently lacking. In practice, adolescents with SAD and other common anxiety disorders are often treated in the same way, with a broad form of Cognitive Behaviour Therapy (CBT). Unfortunately the majority of children and adolescents with SAD continue to meet diagnostic criteria after treatment with broad-based CBT (Evans, Clark, & Leigh, 2021). For example, in one US RCT of generic CBT, recovery among those with SAD was 41% compared to 72% for those with other anxiety disorders (Ginsburg et al., 2011). Therefore, the question arises as to whether the Clark & Wells model is relevant to adolescents, because this would indicate that CT-SAD may be a useful treatment approach. This cannot be assumed given the distinctive neurocognitive, emotional, and social factors in operation at this stage of life (Blakemore, 2008) and many of the processes invoked in the Clark & Wells model are underpinned by cognitive and emotional factors still in development. For example, self-consciousness develops through adolescence and is intimately linked to self-focused attention (Rankin, Lane, Gibbons, & Gerrard, 2004). Addressing this question has the potential to improve outcomes for youth with SAD. Two clinical trials, both of which have compared a broad-based form of CBT with a version of SAD-specific CBT, are relevant (Rapee et al., 2023; Spence, Donovan, March, Kenardy, & Hearn, 2017). Both trials developed an SAD-specific version of CBT that included social skills training, attention training, reduction of safety behaviours, cognitive challenging and coping skills, with neither demonstrating differences between treatment arms at post-treatment or follow-up. Whilst it is possible that this indirectly counters the idea that these processes are implicated in SAD in youth, as suggested by Rapee et al. (2023) and Leigh (2023) it seems more likely that it is because the augmented CBT tested in these two trials does not maximize opportunities to modify maintenance processes.

A number of empirical studies with community samples have tested whether the components of the Clark & Wells model are relevant to adolescents. Cross-sectional observational studies such as those of Hodson, McManus, Clark, and Doll (2008) and Schreiber, Höfling, Stangier, Bohn, and Steil (2012) support an association between negative thoughts, safety behaviours, negative imagery, and self-focused attention and social anxiety symptoms in adolescents (see Leigh and Clark (2018) for a review), and more recently prospective and experimental studies have pointed to a causal role for these processes (Chiu, Clark, & Leigh, 2021; Leigh et al., 2020, 2021). Taken together the findings indicate that targeting these processes in treatment may be beneficial in reducing social anxiety in youth. However, so far, studies have been largely carried out with community rather than clinical populations. Whilst a small number of studies have been undertaken with clinical samples of adolescents (see Leigh and Clark (2018) for a review), to our knowledge none have examined the various processes of interest simultaneously or their specificity to SAD. Therefore, this study aimed to establish whether key cognitive and behavioural processes specified in the Clark and Wells (1995) model are observed in clinical populations and whether they are specific to SAD. To do this, we compared a clinical sample of adolescents with SAD with a clinical sample of adolescents with a non-SAD anxiety disorder and with a community sample of adolescents.

We hypothesized that adolescents with SAD would show significantly higher levels of (i) negative social cognitions and attitudes, (ii) self-focused attention, and (iii) safety behaviours, compared to adolescents with other anxiety disorders and with community adolescents.

## 2 Methods

### 2.1 Study design

The present study was cross-sectional and comprised five self-report questionnaires administered to three groups of adolescents. Questionnaires were selected to measure symptoms of social anxiety, symptoms of anxiety and depression, social cognitions and attitudes, self-focused attention, and safety behaviours.

### 2.2 Participants

Three groups of participants were recruited for the study. The first group (‘SAD’ group) consisted of 90 adolescents with a SAD diagnosis, either as their primary or secondary diagnosis (if secondary, the primary disorder was required to be another anxiety disorder). The second group (‘Anxiety Control’ group) consisted of 58 adolescents with one or more anxiety disorder that was not SAD (i.e., Generalised Anxiety Disorder, Separation Anxiety Disorder, a Specific Phobia, Panic Disorder or Agoraphobia). The SAD and Anxiety Control groups were recruited through a clinical service. The adolescent was required to meet diagnostic criteria for SAD or another anxiety disorder using the ADIS-CP; Silverman & Albano, 1996) and an anxiety disorder had to be identified as the young person's primary problem. The third group (‘Community Control’ group) consisted of 45 adolescents recruited through schools. Due to resourcing limitations, we were not able to conduct diagnostic assessments with this group. Participants in all three groups were required to be aged between 12 and 18 years. They were not eligible to take part if they had an intellectual/learning disability (based on school/clinic/parent information) that may interfere with their ability to read and understand the questions.

### 2.3 Procedure

The study procedures were in accordance with ethical standards and were approved by the National Research Ethics Committee South Central – Berkshire B (reference: 15/SC/0081) and the University of Reading Research Ethics Committee (reference: 15/27). Young people aged 12–15 years provided written assent and their parents/carers provided written consent, while those aged 16–18 years provided written consent.

The two groups with a diagnosed anxiety disorder (SAD and Anxiety Control groups) were recruited through a local NHS-commissioned child and adolescent mental health service between December 2015 and February 2020. To be eligible for referral to the service, young people had to be experiencing symptoms of anxiety and/or depression at a level that was causing distress and/or interference (i.e., likely to meet diagnostic criteria). However, they were not eligible if they had an established autistic spectrum disorder, were taking medication for the treatment of anxiety/depression, had immediate suicidal intent, or had been identified by social care as currently ‘at risk’ due to significant child protection concerns. For those referred, if the referral indicated that they had symptoms of anxiety and were likely to meet the study eligibility criteria, the adolescent and their parent/carer were sent the study information sheets. They then undertook a diagnostic assessment, which involved the young person (and where possible, the parent/carer) being interviewed separately. All assessments were carried out by trained assessors (either clinicians working in the service or placement students, under the supervision of an experienced clinician). All assessors were trained to high levels of reliability and received supervision for every assessment from a clinical psychologist (or equivalent) with extensive experience of delivering and supervising diagnostic assessments and proven reliability. If the diagnostic assessment confirmed that the young person had an anxiety disorder as the main presenting problem, the study was then discussed with them at the subsequent clinical appointment, and they were invited to take part. If the adolescent and their parent agreed, they were then given the set of self-report measures to complete. This could be done as a hard copy or online, through a secure system.

The Community Control group was recruited through schools between March and August 2016. Head teachers were asked to assist with recruitment and if they agreed, the study was advertised through school newsletters and/or invitation letters sent out to students and their parents/carers. For those who indicated they were interested in taking part, adolescents and their parents/carers were sent information sheets about the study. They were then contacted by the research team to address any questions they might have about the study and, if they wished to take part, complete consent/assent forms. Questionnaires could be completed on paper or online. At the end of the questionnaires, participants were told that if they had any concerns or worries to get in touch (or ask their parent/guardian to get in touch) with the research team and were also given further information about support, advice, and resources. Participants in this group were given a £10 gift voucher to reimburse them for their time.

### 2.4 Measures

The measures were given to all participants, except for the diagnostic assessment which was only administered to the SAD and Anxiety Control groups.

**Diagnostic assessment.** The diagnostic assessment was conducted to determine eligibility to the clinical service and the study. It used sections from two validated semi-structured interview schedules to determine whether the young person met diagnostic criteria for an anxiety/depressive disorder; the Anxiety Disorders Interview Schedule (ADIS-IV-C/P; Silverman et al., 1996) (adapted for DSM-5) and the Kiddie Schedule for Affective Disorders and Schizophrenia Present and Lifetime (K-SAD-S-PL; Kaufman et al., 1997). The ADIS was used to assess DSM-5 diagnoses of anxiety and externalising disorders in children and adolescents. It has excellent interrater reliability (Lyneham et al., 2007) and concurrent validity (Wood et al., 2002). In the current study, interrater reliability was good overall (see Supplementary Information for further details). The K-SAD-S-PL was used to assess DSM-V Major and Persistent Depressive Disorder and Mania. It has good concurrent validity of screens and K-SAD-S-PL diagnoses (Kaufman et al., 1997). Both interrater reliability and test-retest reliability are excellent (Kaufman et al., 1997), suggesting that the K-SAD-S-PL produces both reliable and valid psychiatric diagnoses. Using information from both the young person and their parent/carer, the clinician assigns a Clinical Severity Ratings (CSRs) to diagnoses (as is standard practice with the ADIS) using a nine-point scale (0–8), with ‘0’ indicating no impairment and ‘8’ indicating severe impairment. Impairment is defined as affecting the young person's life and/or creating significant distress. A CSR ≥4 signifies a clinical diagnosis. The disorder with the highest CSR was allocated as the primary diagnosis.

**Demographic information.** For all groups, information about the young person (age, gender, ethnicity) was collected.

**Anxiety and depression symptoms**. The Revised Child Anxiety and Depression Scale (RCADS; Chorpita, Yim, Moffitt, Umemoto, and Francis (2000)) is a self-report measure that assesses symptoms of separation anxiety disorder, social anxiety disorder, generalised anxiety disorder, panic disorder, obsessive compulsive disorder and major depressive disorder. There are 47 items and respondents are asked to rate how often each item applies on a scale of 0 (‘never’) to 3 (‘always’). It has been shown to have robust psychometric properties in children and young people from 7 to 18 years of age (Chorpita, Moffitt, & Gray, 2005). For the current study, we used the total RCADS score and all subscale scores. The internal consistency for the total score in our clinical sample (SAD and Anxiety Control participants) and community sample was excellent (clinical sample α = 0.91; community sample α = 0.95). The internal consistency was excellent (α > 0.90) for all subscales in the clinical and community samples.

**Social anxiety symptoms**. The Liebowitz Social Anxiety Scale for Children and Adolescents (LSAS-C/A; Masia, Klein, & Liebowitz, 1999) is a measure of social anxiety symptoms that was used in self-report version for the present study. It includes 24 items, rated on a scale from 0 (‘none’) to 3 (‘severe’) for fear and avoidance of social interaction and performance. The total score includes both the fear and avoidance ratings and scores range from 0 to 144. It has well established psychometric properties when administered to children and young people from 7 to 18 years of age (Leigh & Clark, 2021b; Masia-Warner et al., 2003). The internal consistency for both samples was excellent (clinical sample α = 0.98; community sample α = 0.98).

**Social cognitions.** The Child & Adolescent Social Cognitions Questionnaire (CASCQ; Leigh & Clark, 2021a) is a self-report questionnaire that assesses common social anxiety-related cognitions and was adapted from the adult Social Cognitions Questionnaire (Clark, 2003), for example “People will stare at me” and “People think I am boring”. There are 27 items and respondents are asked firstly to rate how frequently they experience each thought when they are nervous or frightened, from 1 (‘thought never occurs’) to 5 (‘thought always occurs when I am nervous’). They are then asked to rate how much they believe the thought, from 0 (‘I do not believe this thought’) to 100 (‘I am completely convinced this thought is true’). A mean frequency and belief score is calculated. The measure has good internal consistency and convergent validity in an adolescent community sample (Leigh & Clark, 2021a). The internal consistency for both samples was excellent (clinical sample α = 0.97; community sample α = 0.97) for both frequency and belief ratings.

**Social attitudes.** The Child & Adolescent Social Attitudes Questionnaire (CASAQ) is a self-report questionnaire that assesses common social anxiety-related attitudes or beliefs. There are 52 items and respondents are asked how much they agree or disagree with each statement on a 7-point scale from ‘totally agree’ to ‘totally disagree’. A mean CASAQ score is calculated, with lower scores indicating a more negative attitude. The measure was originally developed for adults and has good internal consistency and discriminant validity (Clark, 2003). The internal consistency for both samples was excellent (clinical sample α = 0.92; community sample α = 0.93).

**Self-focused attention**. Two items from the Child and Adolescent Social Summary Weekly Rating Scale (CASSWRS) (Clark, 2003) were used to measure self-focused attention over the previous week. One item assessed self-focused attention for social situations that the participant found difficult (“For social situations that you found difficult, please choose a number from the scale below to show how much your mind was focused on yourself (‘self-focused’) or on other people and what was going on around you (‘externally focused’) in the last week”). The other item assessed self-focused attention for social situations in general (“For social situations in general, please choose a number from the scale below to show how much your mind was focused on yourself (‘self-focused’) or on other people and what was going on around you (‘externally focused’) in the last week”). Both items are scored from 0 (‘entirely externally focused’) to 8 (‘entirely self-focused’), and a mean is obtained. The internal consistency for our clinical sample was acceptable (α = 0.76) but for our community sample it was questionable (α = 0.65).

**Social behaviours**. The Child and Adolescent Social Behaviour Questionnaire (CASBQ; Evans, Chiu, Clark, Waite, & Leigh, 2021) is a self-report questionnaire consisting of 28 items that reflect a range of safety behaviours in social situations. For each item, the respondent is asked to rate how often they undertake the behaviour when they are anxious in or before a social situation, from 0 (‘never’) to 3 (‘always’). An overall CASBQ score is calculated as a mean of responses, with a range of 0 (no safety behaviours) to 3 (very frequent safety behaviours). The original version of the questionnaire designed for use with adults (Social Behaviour Questionnaire; Clark (2003)) has good psychometrics properties (Clark, 2005). Sound psychometric properties of the youth version have been found with adolescents (Evans, Chiu, Clark, Waite, & Leigh, 2021). The internal consistency was good in our clinical sample (α = 0.89) and excellent in our community sample (α = 0.92).

### 2.5 Analysis plan

Analysis was undertaken in SPSS and R version 2022.02.3. Mean substitution was performed when less than 10% of items were missing in each questionnaire. Questionnaires with more than 10% of missing items were treated as missing variables at participant level with complete cases used in the analysis. For the clinical sample, at variable level, 5% of data was missing for the RCADS total, 8% for the LSAS-CA, 8% for the CASCQ-F, 11% for the CASCQ-B, 5% for the CASBQ, 9% for the CASAQ and 7% for the CASPWSS. For the community sample, 4% of data was missing for the RCADS total, 0% for the LSAS-CA, 2% for the CASCQ-F, 2% for the CASCQ-B, 2% for the CASBQ, 0% for the CASAQ and 0% for the CASPWSS.

Descriptive statistics are reported. A series of ANCOVA were undertaken with Group (SAD vs. Anxiety Control and SAD vs. Community Control) as between-subjects factors, and age and gender as covariates were undertaken to examine differences in symptom scores and process scores. We ran separate ANCOVAs to compare the SAD group to the Anxiety Control group and then the SAD group to the Community Control group because we did not compare the two control groups directly. We report generalised eta-square as an estimate of the effect size.

## 3 Results

### 3.1 Descriptive statistics

For the 90 participants in the SAD group, this was the primary problem for 62% of participants [n = 56] and the secondary problem for 38% [n = 34] (amongst these participants, GAD was the most common primary problem). 90.47% of the SAD group were White British. For the 58 participants in the Anxiety Control group, the three most common primary diagnoses were: GAD (n = 19; 33%), Separation Anxiety Disorder (n = 13; 22%), and specific phobia (n = 13; 22%). 79.31% of this group were White British. Data on the ethnicity of the Community sample was not available.

Table 1 provides demographic and clinical information on the participants. Comparing the SAD group and Community Control group, the SAD group had significantly more girls (χ ^2^(1) = 17.60, *p* < .001) and was significantly younger (*t*(133) = 2.00, *p* = .024. The SAD and Anxiety Control groups did not significantly differ in terms of gender (χ^2^(1) = 0.34, *p* = .560), but the SAD group was significantly older than the Anxiety Control group (*t*(146) = 4.14, *p* < .001).

**Table 1:** Descriptive statistics for SAD and anxiety control clinical groups and community control group.

	SAD	Anxiety control	Community control
Total N	90	58	45
Gender, n (%)			
Girls/young women	69 (76.7%)	42 (72.4%)	18 (40.0%)
Boys/young men	21 (23.3%)	16 (27.6%)	27 (60.0%)
Non-binary	0	0	0
Age Mean (SD)	14.06 (2.27)	12.40 (2.73)	14.89 (2.30)
Primary anxiety disorder, n (%)			
SAD	56 (62.2%)	0	–
GAD	26 (28.9%)	19 (32.8%)	–
Separation anxiety disorder	3 (3.3%)	13 (22.4%)	–
Specific phobia	2 (2.2%)	13 (22.4%)	–
Panic disorder	1 (1.1%)	6 (10.3%)	–
Agoraphobia	1 (1.1%)	5 (8.6%)	–
Illness anxiety disorder	1 (1.1%)	0	–
AD-NOS	0	2 (3.4%)	–
Presence of diagnoses, n (%)			
SAD	90 (100%)	0	–
GAD	70 (77.8%)	30 (51.7%)	–
Separation anxiety disorder	15 (16.7%)	18 (31.0%)	–
Specific phobia	13 (14.4%)	18 (31.0%)	–
Panic disorder	10 (11.1%)	7 (12.1%)	–
Agoraphobia	10 (11.1%)	9 (15.5%)	–
Illness anxiety disorder	1 (1.1%)	0	–
AD-NOS	0	3 (5.2%)	–
OCD	2 (2.2%)	1 (1.7%)	–
PTSD	1 (1.1%)	0	–
Dysthymia	3 (3.3%)	0	–
MDD	5 (5.6%)	3 (5.2%)	–
ADIS reporter, n (%)			
Adolescent + parent report	81(90.0%)	54 (93.1%)	
Adolescent-only report	9 (10.0%)	4 (6.9%)	

Notes: AD-NOS = anxiety disorder – not otherwise specified; GAD = generalised anxiety disorder; MDD = major depressive disorder; OCD = obsessive compulsive disorder; PTSD = post-traumatic stress disorder; SAD = social anxiety disorder; SD = standard deviation.

### 3.2 Symptom measures: group comparisons

Symptom measures for the SAD, Anxiety Control, and Community Control groups are shown in Table 2 along with the ANCOVA test statistics. The SAD group scored significantly higher than the Anxiety Control group on both measures of social anxiety symptoms and on the RCADS total scale, with no differences between the groups on the other subscales of the RCADS. The SAD group scored significantly higher than the Community Control group on all symptom measures.^1^ ^2^

**Table 2:** Symptom and process measure scores for SAD and Anxiety Control Clinic Samples and Community Sample.

	SAD Mean (SD)	Anxiety Controls Mean (SD)	Statistical Test SAD vs Anxiety Controls^†^	η^2^G	Community Controls Mean (SD)	Statistical Test SAD vs Community Controls^†^	η^2^G
Symptom Measures							
RCADS-Total	**67.33 (22.17)**	53.58 (21.02)	F(1, 132) = 8.33, *p* < .01	0.059	35.36 (20.35)	F(1, 124) = 41.25, *p* < .001	0.250
RCADS-MDD	**13.35 (5.38)**	10.45 (6.22)	F(1, 132) = 2.26, *p* = .135	0.017	7.95 (5.76)	F(1, 124) = 20.56, *p* < .001	0.142
RCADS-GAD	**9.32 (3.63)**	8.76 (3.89)	F(1, 131) = 0.46, *p* = .497	0.004	5.74 (3.33)	F(1, 123) = 15.43, *p* < .001	0.111
RCADS-SEP	**8.02 (4.09)**	7.78 (4.86)	F(1, 131) = 2.33, *p* = .129	0.017	2.37 (2.41)	F(1, 123) = 45.00, *p* < .001	0.268
RCADS-PD	**11.53 (5.92)**	10.12 (6.06)	F(1, 132) = 0.25, *p* = .621	0.002	4.64 (4.36)	F(1, 124) = 27.45, *p* < .001	0.181
RCADS-OCD	**5.64 (3.64)**	5.59 (3.38)	F(1, 131) = 0.21, *p* = .648	0.002	3.84 (3.24)	F(1, 123) = 4.68, *p* < .05	0.037
RCADS-SAD	**19.32 (6.57)**	10.881 (6.65)	F(1, 132) = 35.61, *p* < .001	0.212	10.86 (6.20)	F(1, 124) = 38.71, *p* < .001	0.238
LSAS-CA	**87.92 (30.88)**	39.35 (29.30)	F(1, 130) = 62.78, *p* < .001	0.326	50.70 (35.18)	F(1, 125) = 33.81, *p* < .001	0.213
Process Measures							
CASCQ-F	**2.95 (0.85)**	1.96 (0.91)	F(1, 131) = 27.92, *p* < .001	0.172	2.22 (0.90)	F(1, 122) = 11.85 *p* < .001	0.089
CASCQ-B	**47.69 (21.54)**	21.87 (21.40)	F(1, 125) = 32.59, *p* < .001	0.207	27.60 (24.35)	F(1, 119) = 14.96, *p* < .001	0.112
CASAQ	**2.18 (0.83)**	3.29 (1.13)	F(1, 103) = 24.52, *p* < .001	0.192	3.27 (1.09)	F(1, 113) = 24.01, *p* < .001	0.175
SFA	**4.07 (1.87)**	3.27 (1.73)	F(1, 131) = 2.99, *p* < .05	0.032	4.10 (1.42)	F(1, 123) = 0.11, *p* = .744	0.001
CASBQ	**1.39 (0.43)**	0.83 (0.58)	F(1, 134) = 27.31, *p* < .001	0.169	1.03 (0.50)	F(1, 125) = 15.61, *p* < .001	0.111

Notes: CASAQ = Child & Adolescent Social Attitudes Questionnaire; CASCQ-F = Child & Adolescent Social Cognitions Questionnaire - Frequency; CASCQ-B = Child & Adolescent Social Cognitions Questionnaire – Belief; CASAQ = Child & Adolescent Social Attitudes Questionnaires; SFA = Child and Adolescent Social Summary Weekly Rating Scale – Self-Focused Attention items; LSAS-CA The Liebowitz Social Anxiety Scale for Children and Adolescents; RCADS-Total = The Revised Child Anxiety and Depression Scale – Total Score; RCADS-SAD = The Revised Child Anxiety and Depression Scale – Social Anxiety Disorder Subscale Score; SAD = social anxiety disorder; MDD = depression; SEP = separation anxiety disorder; GAD = generalised anxiety disorder; PD = panic disorder; OCD = obsessive compulsive disorder; SD = standard deviation; η^2^G = generalised eta squared; ^†^ One way ANCOVA controlling for age and sex.

### 3.3 Process measures: group comparisons

**Fig. 1:**
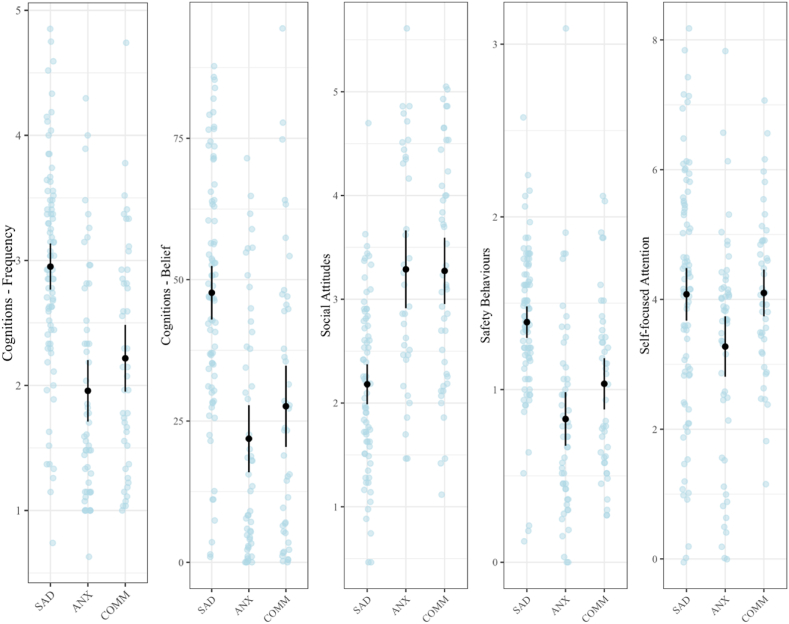
Process Measure Scores for SAD Clinical Group, Anxiety Control Group and Community Control Group (means with 95% Confidence Intervals displayed).

Mean and standard deviations for the cognitive and behavioural process measures in the SAD, Anxiety Control, and Community Control groups are shown in Tables 2 and in Fig. 1.

The ANCOVA test statistics are shown in Table 2. As can be seen, measures of negative social cognitions (CASCQ both frequency and belief ratings) and safety behaviours (CASBQ) were significantly higher in the SAD group compared to both the Anxiety and Community Control samples. Similarly, social attitudes scores (CASAQ) were significantly lower (more negative) in the SAD group compared to both the Anxiety and Community Control samples. Self-focused attention was significantly higher in the SAD group compared to the Anxiety Control group but not the Community Control group.^1^, ^2^, ^3^

## 4 Discussion

This study aimed to examine whether key cognitive and behavioural processes specified in the Clark and Wells (1995) model of SAD (in adults) are observed in adolescents. It builds on existing studies by examining this in clinical and community populations and determining the extent to which the processes are specific to SAD. Consistent with our hypotheses, we found that adolescents with SAD showed significantly more negative social cognitions and social attitudes and more safety behaviours compared to adolescents with other anxiety disorders and with community adolescents. Self-focused attention was significantly higher in the SAD group than the anxiety control group, but contrary to our hypothesis it was not significantly higher in the SAD group compared to the community control group.

Our finding that adolescents with SAD showed significantly more negative social cognitions, social attitudes and more safety behaviours compared to adolescents with other anxiety disorders and with community adolescents is consistent with previous literature and promising findings from recent clinical trials using treatments based on Clark and Wells’ cognitive model. Studies with mainly community-based adolescents have shown associations between negative thoughts, safety behaviours, and self-focused attention, and social anxiety symptoms using cross-sectional (e.g., Hodson et al. (2008); Schreiber et al. (2012)) as well as prospective and experimental designs (Chiu et al., 2021; Leigh et al., 2020, 2021). Taken together, these findings indicate that targeting these processes in treatment for adolescent SAD is likely to be beneficial. This is indeed borne out by the findings of two trials (Ingul, Aune, & Nordahl, 2014; Leigh & Clark, 2023), which have demonstrated that CT-SAD is associated with large controlled treatment effects and furthermore the trial of Leigh & Clark (2022) found that the beneficial effect of CT-SAD was mediated by changes in these processes.

Although the SAD group endorsed more self-focused attention in social situations compared to the Anxiety Control group, we were surprised to find that there was no significant difference between the SAD group and the Community group. A number of studies with community samples have reported an association between self-focused attention and social anxiety symptoms (Blote, Miers, Heyne, Clark, & Westenberg, 2014; Chiu et al., 2021; Hodson et al., 2008; Leigh et al., 2021; Schreiber et al., 2012). It may be that there is no difference in self-focused attention between these groups, or it could be that it is a mechanism relevant across anxiety disorders more broadly. Alternatively, the failure to find an effect may in part be methodological. We created an average of two items (taken from the SPWSS) to measure self-focused attention. However, the internal consistency for the measure in the Community group was below the accepted level of 0.7 (Nunally & Bernstein, 1978). Measures with one or two items are prone to random measurement errors and may underestimate associations and so it is possible that this methodological limitation explains the finding. Use of a multi-item measure such as the Focus of Attention Questionnaire (Woody, Chambless, & Glass, 1997) to capture self-focused attention will increase reliability and mitigate against this limitation in future studies.

The findings add to the existing evidence base and suggests that not only are these cognitive and behavioural processes sensitive markers of SAD but they are also specific SAD process variables. With this study we addressed what Garber and Hollon (1991) called the question of broad specificity, in that we examined whether these cognitive and behavioural processes could differentiate young people with SAD from those with a range of other anxiety problems. It will be informative to now examine the question of narrow specificity, or in other words whether the processes can differentiate SAD from other specific anxiety disorders such as compared to those with generalised anxiety disorder or panic disorder.

Our findings indicate that the core processes that are implicated in the maintenance of adult SAD are also relevant to adolescents. However we note the broad age range assessed in the present study which precludes a fine-grained analysis of how these processes vary across the adolescent period. But this would be a valuable avenue of future research because recent studies suggest that there may be differences. For example, it was found that the types of safety behaviours used differed between younger and older adolescents, with increasing use of more cognitively sophisticated behaviours with age (Evans, Chiu, et al., 2021).

The findings have important clinical implications. They help us to understand why outcomes for adolescents with SAD from traditional broad-based anxiety treatments may be disappointing and point to the value of disorder specific interventions for this population. They also indicate that we may achieve better outcomes by carefully targeting these processes in therapy and this is best done in an integrated fashion (Leigh, 2023). One such treatment is Cognitive Therapy. Cognitive Therapy is comprised of a series of techniques addressing negative social cognitions and beliefs, self-focused attention, and unhelpful safety behaviours. Core techniques include video feedback and behavioural experiments. In support of this assertion, two randomised controlled trials with adolescents with 10.13039/501100009237SAD have found the treatment to be associated with large controlled effect sizes (Ingul et al., 2014; Leigh & Clark, 2023).

This study has strengths. It examines maintenance mechanisms in clinical samples that allow us to understand the extent to which they are distinct to SAD. Adolescents in the clinical groups were all referred for treatment within routine services and all received a gold standard diagnostic assessment, conducted by assessors with high levels of training and supervision. In terms of limitations, we were not able to carry out diagnostic assessments with the group of adolescents recruited from the community. Although, as expected, the group's mean scores on all symptom measures were significantly lower than those of the SAD group, it is possible that this group included young people who met diagnostic criteria for an anxiety disorder and potentially other disorders. However, the fact that we found the differences between the groups with this more conservative bar makes the findings potentially more robust. Because we wanted to examine the extent to which differences between the two clinical groups was accounted for by SAD, our Anxiety Control group did not include anyone with SAD. It is important to note that as a result this group is not a representative clinical sample because SAD is common as both a primary and secondary anxiety disorder in adolescents.

We note minor demographic differences between groups; the SAD group included significantly more girls than the Community Control group. On average the SAD group was significantly older than the Anxiety Control group and younger than the Community Control group. However, both variables were controlled for in the analysis. We were able to collect ethnicity data for our two clinical groups, and it is important to acknowledge that the SAD sample was predominantly White British and therefore findings may not be generalisable to more diverse groups. In addition, we were not able to collect ethnicity data from the community control group and therefore there may be important differences that are not accounted for.

To conclude, the findings from this study establish that key cognitive and behavioural processes specified in the Clark and Wells (1995) model are observed in adolescent clinical populations and support the suggestion that they are specific to 10.13039/501100009237SAD. Historically, adolescent SAD has been associated with poorer treatment outcomes. Our findings add to the growing evidence base that suggests that interventions that single-mindedly target these cognitive and behavioural processes may yield better outcomes for this population.

## CRediT authorship contribution statement

**Eleanor Leigh:** Conceptualization, Methodology, Formal analysis, Writing – original draft. **Ray Percy:** Investigation, Writing – review & editing. **David M. Clark:** Conceptualization, Methodology, Writing – review & editing. **Cathy Creswell:** Conceptualization, Methodology, Writing – review & editing. **Polly Waite:** Conceptualization, Methodology, Formal analysis, Writing – original draft.

## Declaration of competing interest

None

## Data Availability

Data will be made available on request.

## Footnotes

1 Results remained unchanged when we applied the Benjamini Hochberg method with a 10% false discovery rate to control for Type 1 errors due to multiple comparisons.

2 As a sensitivity analysis, analyses were rerun with a primary SAD only SAD group (i.e., participants with a secondary diagnosis of SAD were excluded). Results are reported in the Supplementary Information.

3 ANCOVAs were rerun with depression symptoms (as measured by the child reported RCADS depression subscale) as an additional covariate. There were no differences in the pattern of results.
