# Supplementary material for: Cognitive and behavioural processes in adolescents with social anxiety disorder
Source: Behav Res Ther. 2023 Nov;170:104416. doi: 10.1016/j.brat.2023.104416 (PMC10933732; doi:10.1016/j.brat.2023.104416)
Supplement: Multimedia component 1 [file mmc1.docx]

SUPPLEMENTARY INFORMATION

ADIS-CA Interviews Interrater Reliability

A sub-sample of 85 assessments (57%) was reviewed to check the level of agreement between provisional diagnoses and CRSs assigned by assessors and those agreed at consensus meetings with a supervisor. Interrater reliability was good for SAD, GAD, separation anxiety disorder, specific phobia, panic disorder, and agoraphobia (diagnosis: kappa = .79 to .93; CSR: weighted kappa = .78 to .90), and these conditions accounted for 94% (290) of all diagnoses. Interrater reliability for illness anxiety disorder, ADNOS, OCD, PTSD, dysthymia, and MDD – which together accounted for just 6% (19) of all diagnoses – was below accepted levels (diagnosis: kappa = .26 to .66; CSR: weighted kappa = .32 to .66). Because these conditions occurred rarely (see Supplementary Table 1), any disagreement between raters inevitably had a large impact on reliability statistics. Overall, therefore, interrater reliability should be considered good.

Supplementary Table 1

|  | **Diagnosis (Kappa)** | **CSR (weighted Kappa)** |
| --- | --- | --- |
| Social anxiety disorder | .87 | .85 |
| GAD | .78 | .79 |
| Separation anxiety disorder | .81 | .81 |
| Specific phobia | .93 | .90 |
| Panic disorder without agoraphobia | .85 | .87 |
| Panic disorder with agoraphobia | .90 | .90 |
| Agoraphobia without panic disorder | .79 | .78 |
| Illness anxiety disorder | -- | -- |
| ADNOS (1x instance) | .66 | .57 |
| OCD (3x instances) | .49 | .37 |
| PTSD (0x instances) | -- | -- |
| Persistent depressive disorder (2x instances) | .66 | .66 |
| Major depressive disorder (4x instances) | .26 | .32 |

Sensitivity Analysis

The ANCOVA analyses were repeated with the SAD group defined to include only those with a primary diagnosis of SAD (i.e., those with a secondary diagnosis of SAD were excluded). 57 adolescents had a primary diagnosis of SAD.

The means and standard deviations for the primary SAD group on symptom and process measures as well as the results of the ANCOVAs are shown in Supplementary Table 2.

The same pattern of results was observed in the sensitivity analysis as in the main analysis with one exception. In the sensitivity analysis, there was no significant difference between the SAD and community control groups on the OCD subscale of the RCADS measure whereas a difference was found in the main analysis.

Supplementary Table 2

|  | **SAD**  **Mean (SD)** | Statistical Test  SAD vs Anxiety Controls^†^ | η^2^G | Statistical Test  SAD vs Community Controls^†^ | η^2^G |
| --- | --- | --- | --- | --- | --- |
| Symptom Measures | | | | | |
| RCADS-Total | **66.04 (21.53)** | F(1, 102) = 4.48, *p*<.05 | 0.042 | F(1, 94) = 33.66, *p*<.001 | 0.264 |
| RCADS-MDD | **13.65 (5.59)** | F(1, 102) = 0.92, *p*=.340 | 0.009 | F(1, 94) = 16.78, *p*<.001 | 0.151 |
| RCADS-GAD | **9.07 (3.76)** | F(1, 102) = 0.07, *p*=.788 | 0.001 | F(1, 94) = 11.15, *p*<.001 | 0.106 |
| RCADS-SEP | **7.44 (3.94)** | F(1, 102) = 0.68, *p*=.411 | 0.007 | F(1, 94) = 36.92, *p*<.001 | 0.282 |
| RCADS-PD | **11.32 (5.74)** | F(1, 102) <0.01, *p*=.954 | <0.001 | F(1, 94) = 25.27, *p*<.001 | 0.212 |
| RCADS-OCD | **5.26 (3.50)** | F(1, 101) <0.01, *p*=.993 | <0.001 | F(1, 93) = 2.71, *p*=.103 | 0.037 |
| RCADS-SAD | **19.27 (5.14)** | F(1, 102) = 32.74, *p*<.001 | 0.243 | F(1, 94) = 38.37, *p*<.001 | 0.290 |
| LSAS-CA | **96.10 (27.56)** | F(1, 99) = 79.43, *p*<.001 | 0.445 | F(1, 125) = 37.97, *p*<.001 | 0.288 |
| Process Measures | | | | | |
| CASCQ-F | **3.02 (0.94)** | F(1, 101) = 18.98, *p*<.001 | 0.158 | F(1, 92) = 8.69, *p*<.01 | 0.086 |
| CASCQ-B | **51.40 (22.56)** | F(1, 95) = 31.28, *p*<.001 | 0.248 | F(1, 89) = 14.35, *p*<.001 | 0.139 |
| CASAQ | **2.11 (0.89)** | F(1, 80) = 20.69, *p*<.001 | 0.205 | F(1, 90) = 19.92, *p*<.001 | 0.181 |
| SFA | **4.48 (1.88)** | F(1, 101) = 5.40, *p*<.05 | 0.051 | F(1, 93) = 0.96, *p*=.331 | 0.010 |
| CASBQ | **1.47 (0.42)** | F(1, 103) = 23.65, *p*<.001 | 0.187 | F(1, 94) = 15.76, *p*<.001 | 0.144 |

Notes: CASAQ = Child & Adolescent Social Attitudes Questionnaire; CASCQ-F = Child & Adolescent Social Cognitions Questionnaire - Frequency; CASCQ-B = Child & Adolescent Social Cognitions Questionnaire – Belief; CASAQ = Child & Adolescent Social Attitudes Questionnaires; SFA = Child and Adolescent Social Summary Weekly Rating Scale – Self-Focused Attention items; LSAS-CA The Liebowitz Social Anxiety Scale for Children and Adolescents; RCADS-Total = The Revised Child Anxiety and Depression Scale – Total Score; RCADS-SAD = The Revised Child Anxiety and Depression Scale – Social Anxiety Disorder Subscale Score; SAD = social anxiety disorder; MDD = depression; SEP = separation anxiety disorder; GAD = generalised anxiety disorder; PD = panic disorder; OCD = obsessive compulsive disorder; SD = standard deviation; η^2^G = generalised eta squared; ^†^ One way ANCOVA controlling for age and sex.
